# Achieving clinical success with BET inhibitors as anti-cancer agents

**DOI:** 10.1038/s41416-021-01321-0

**Published:** 2021-03-15

**Authors:** Tatiana Shorstova, William D. Foulkes, Michael Witcher

**Affiliations:** 1 Departments of Oncology and Experimental Medicine, McGill University, Lady Davis Institute and Segal Cancer Centre, Jewish General Hospital, Montreal, QC Canada; 2 Departments of Oncology and Human Genetics, McGill University, Lady Davis Institute and Segal Cancer Centre, Jewish General Hospital, Montreal, QC Canada

**Keywords:** Predictive markers, Targeted therapies

## Abstract

The transcriptional upregulation of oncogenes is a driving force behind the progression of many tumours. However, until a decade ago, the concept of ‘switching off’ these oncogenic pathways represented a formidable challenge. Research has revealed that members of the bromo- and extra-terminal domain (BET) motif family are key activators of oncogenic networks in a spectrum of cancers; their function depends on their recruitment to chromatin through two bromodomains (BD1 and BD2). The advent of potent inhibitors of BET proteins (BETi), which target either one or both bromodomains, represents an important step towards the goal of suppressing oncogenic networks within tumours. Here, we discuss the biology of BET proteins, advances in BETi design and highlight potential biomarkers predicting their activity. We also outline the logic of incorporating BETi into combination therapies to enhance its efficacy. We suggest that understanding mechanisms of activity, defining predictive biomarkers and identifying potent synergies represents a roadmap for clinical success using BETi.

## Background

Cancer is a heterogeneous disease that is characterised by abnormal cell proliferation and a range of molecular defects acquired during tumorigenesis. These renowned ‘hallmarks of cancer’ include sustained proliferative signalling, insensitivity to growth-suppressive signals, resistance to apoptosis, replicative immortality, angiogenesis, the capacity to invade and metastasise, dysregulation of energy metabolism and the avoidance of immune detection.^1^ Inflammation driven by cytokine release from tumours, genome instability and mutation are also tumour-enabling characteristics.

Appropriately controlled regulation of gene expression is critical for homoeostasis and genome stability, and an important aspect of tumour biology that falls within the hallmark of genome instability is transcriptional dysregulation. Dysregulated transcriptional programmes trigger two major events that can promote cancer: the activation of oncogenes and, conversely, the silencing of tumour suppressor genes. Both of these processes can subsequently affect multiple cancer hallmarks, thereby influencing both cancer initiation and progression. To translate this biological concept into relevant clinical interventions, it is important to identify master transcriptional regulators that drive these diverse oncogenic networks in order to pinpoint nodes for therapeutic intervention.

**Fig. 1:**
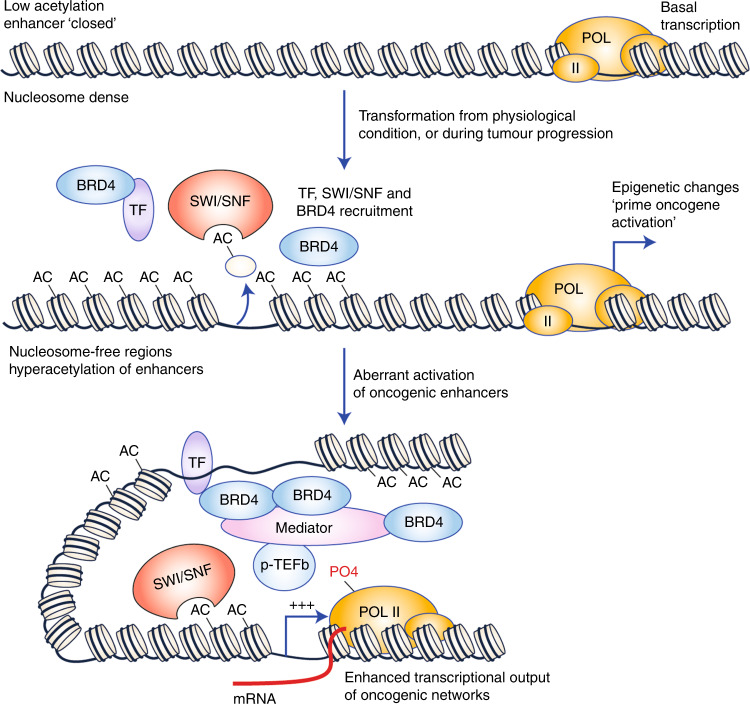
Transcriptional activation of oncogenes by BRD4. Under physiological conditions, proliferation and survival genes are transcribed at a basal rate to maintain homoeostasis. During the transformation of normal epithelium to neoplasia or, similarly, during the progression of a primary tumour to a more invasive stage, chromatin surrounding proto-oncogenes becomes enriched for histone acetylation, especially at enhancer regions. This change in chromatin programming allows nucleosome decompaction, which facilitates the recruitment of bromodomain chromatin remodellers, such as the SWI/SNF complex, that further open chromatin to allow the binding of transcription factors (TF). Acetylation also facilitates the recruitment of bromodomain-carrying coactivators, such as BRD4. BRD4 potently activates transcription through the recruitment of the Mediator complex, which connects enhancer elements with the RNA POLII complex at the promoter region of proto-oncogenes. Mediator, through association with CDK9, a component of the p-TEFb elongation complex, phosphorylates RNA POL II on serine 2 of its C-terminal domain, thereby stimulating transcriptional elongation.

Chromatin modifications, such as the post-translational modification of histones and DNA methylation, establish a connection between repressive or permissive chromatin structure and transcriptional outputs.^2,3^ Understanding the influence of dysregulated epigenetic modification on transcriptional outputs and using this information to uncover novel therapeutic avenues to treat cancer has been an important research goal for several decades.^4,5^ It is clear that dysregulation of many modifications including both transcriptional activating or repressing marks leads to aberrant transcriptional outputs such as heightened expression of oncogenes. One classical example of this is the accumulation of transcriptionally activating lysine acetylation at enhancer regions of oncogenes such as *c-MYC*.^6^ The acetylation of multiple lysine residues within the N-terminal tail of core histones, mediated by histone acetyltransferases (HATs), can be recognised by proteins carrying bromodomains (BRDs),^7–9^ which generally increase the rate of transcription of associated genes (Fig. 1)^10^ through diverse mechanisms including the recruitment of transcriptional complexes and chromatin remodelling.

Based on the crystal structure, BRD proteins can be categorised into eight families.^11^ One important BRD subfamily includes the bromo- and extra-terminal domain (BET) proteins—BRD2, BRD3, BRD4 and BRDT. BET proteins recognise histone acetylation through one of their two tandem bromodomains, BD1 or BD2, and activate transcription through recruitment of the multiprotein mediator complex and positive transcription elongation factor b (P-TEFb), thereby enhancing transcriptional elongation.^12,13^ BD1 and BD2 recognise distinct sets of acetylated histones. For example, the BD1 pocket of BRD4 has a strong preference for combinations of acetylation marks on histone 4, but shows a weaker affinity for acetylated lysine residues on histone 3.^11^ Evidence suggests that multiple BET-family members might be required for the rapid induction of target genes,^14^ which suggests that the members might have non-overlapping functions or perhaps work together within a complex.

As BET proteins are important regulators of transcriptional outputs, it is not surprising that this family of proteins has important roles in homoeostasis and cell survival, and that their dysregulation can promote cancer. Consequently, attempts have been made to synthesise inhibitors of these proteins—BET inhibitors (BETi)—as therapeutic agents that restore appropriately regulated gene expression. We begin this review by outlining the involvement of BET-family members in the hallmarks of cancer, especially avoiding growth suppression and resisting cell death. Subsequently, we will introduce inhibitors of BET-family members, elaborating on biomarkers predicting their efficacy, mechanisms of resistance and enhancing their potency through the use of combination therapies.

## A role for BET proteins in cancer

BET-family members influence cell cycle progression by activating oncogenes such as *MYC*, *JUNB*, *CCND1* and *CCNA1*.^15–17^ Consistent with a role in regulating the expression of cell cycle genes, knockdown of BRD4 leads to arrest in S phase in some cell types.^17^ BET proteins may directly activate oncogene transcription through recruitment to hyper-acetylated regulatory regions, as is seen is the regions surrounding *MYC* and *CCNA1* genes. Upon being recruited to chromatin, BET-family members engage the mediator complex, which in turn, interacts with the core transcription machinery. Mediator provides a platform for the association of the pTEFb complex, leading to the phosphorylation of RNA POL II on serine 2, acting as a catalyst for transcriptional elongation.

Cell cycle genes are controlled, in part, by the E2F family of transcription factors. BRD4 binds strongly to the regulatory regions of E2F1 transcriptional targets to enhance their activation, thereby promoting cell cycle progression.^18,19^ Interestingly, BRD2 may associate with E2F1 and influence its targeting to regulatory regions.^16,20^

Oncogenic roles for BRD4 and BRD3 were first revealed from their propensity to translocate, forming fusion proteins with NUT (nuclear protein in testis, also known as NUTM1). The BRD4–NUT fusion (t(15;19)) is highly oncogenic and initiates the development of NUT-midline carcinoma (NMC), an aggressive tumour with a very poor prognosis.^21^ The driving oncogenic nature of this translocation was confirmed by whole-exome sequencing, in which BRD4–NUT appears as a unique genomic aberration.^22^ Furthermore, inhibiting BRD proteins (discussed below) reduced tumour cell proliferation and contributed to squamous cell differentiation and apoptosis.^23^ BRD4–NUT leads to the activation of pro-survival genes such as *MYC*, which maintains NMC cells in an undifferentiated, proliferative state.^24^

As well as the *BRD4–NUT* fusion event disrupting normal BRD4 function,^25^ it is clear that BRD4 overexpression alone in some contexts might be oncogenic. This shift from maintaining homoeostasis to promoting proliferation probably arises from the mistargeting of BRD4 to the regulatory regions of oncogenes due to changes in histone acetylation. Studies using BETi indicate this role might be dependent on the recruitment of BET-family members to super-enhancers—large genomic regions containing several enhancers in close proximity—where they aberrantly activate oncogenes.^10^ Among the BET-family members, the overexpression of BRD4, in particular, appears to transcriptionally activate target genes that play key roles in cell cycle progression and survival/repression of apoptosis across cancer types. A short hairpin (sh)RNA screen revealed that BRD4 is essential for the proliferation of ovarian carcinomas and that BRD4 depletion significantly reduced cancer cell viability.^26^ Similarly, BRD4 upregulation has been found in renal cell carcinoma, and subsequent inhibition of its expression with shRNAs induced cell cycle arrest.^27^ BRD3 and BRD4 promote cell cycle progression and resistance to apoptosis in cancer by upregulating anti-apoptotic family members including BCL-2 and the cyclin-dependent kinase CDK6.^28^

In addition, BRD4 has been implicated in cell invasion and migration—in a breast cancer model, BRD4 inhibition abrogated the invasion of breast cancer cells and downregulated the expression of Snail, a transcription factor involved in the process of epithelial–mesenchymal transition.^29^ BET proteins have also been long recognised to transcriptionally control inflammatory responses.^30^ BET-family members appear to work co-operatively to control the release of pro-inflammatory cytokines from macrophages.^31^ BETi were demonstrated to suppress the release of a panel of cytokines from macrophages after stimulation using lipopolysaccharide, and BRD4 might act as a co-activator of transcription mediated by the pro-inflammatory molecule nuclear factor κB (NF-κB).^32,33^ BET proteins also potentiate cytokine release from tumours cells.^14^ Considering the important role of immune cells within the tumour microenvironment in facilitating tumour growth and metastasis through cytokine release, it is likely that BET proteins contribute to this hallmark of cancer. As a logical extension of this concept, targeting BET proteins might influence the tumour microenvironment and tumour growth by suppressing pro-inflammatory cytokine release from macrophages within the tumour niche, and the tumour themselves.

To more thoroughly understand the role of BET proteins in tumour progression, it will be necessary to evaluate the impact of elevating the expression of each BET-family member either alone, or in combination, and to subsequently define their relative contribution to various oncogenic processes. Currently, it is unclear whether all family members contribute equally to neoplastic growth, and whether selectively targeting a subset of family members will result in compensation by active, untargeted, BET proteins. While BET proteins are commonly overexpressed in cancer, it remains unproven that they act as oncogenic drivers in all cases. Tumour cells rely strongly on upregulation of oncogenes to sustain their growth and survival, and the elevation of BET-family members may not be oncogenic per se, but act as a ‘non-oncogene addiction’, facilitating widespread transcriptional activation of critical oncogenes. Further studies are warranted to define the cancer subtypes in which BET proteins act as tumour promoters as opposed to a non-oncogene addiction.

## Small molecule inhibitors of BET proteins

The intensive development of BETi first gained traction in 2010 upon the successful synthesis of two structurally related molecules^34^ that competitively bind the acetyl-lysine recognition motif (bromodomain) of multiple BET proteins^23,30,35–39^ (Table 1). We will discuss these two molecules, JQ1 and I-BET762 (GSK525762A, molibresib, I-BET), at length, followed by a description of recent advances in the development of novel BETi.

**Table 1:** BET inhibitors.

Compound	Typical in vitro concentration (µmol/l)	Typical range of in vivo dosage (mg/kg)	*C*_max_	Reference(s)
JQ1	∼0.3–1	30–50	1.18–11 µg/ml	^23,40,44–46,88,120^
I-BET762	∼0.2–1	25–30	∼10 µmol/l	^28,30,36,37,54,55^
OTX015	∼0.3–1	20–50	1.36 µg/ml	^47,57,88^
I-BET151	∼0.3–1	30–50	>10 µmol/l	^28,35,49,55^
RVX-208	∼5	15–60		^38,67^
MS417	∼0.2–1	0.08–20		^73–75,77^
ABBV-075	∼0.01–0.1	0.5–2	∼10 µmol/l	^64,77,109^
ABBV-744	<0.1–1	4.7		^64^
SJ432	∼0.02–0.5	5–15	>4 µg/ml	^66^
AZD5153	∼0.01–0.1	3.6–12.8	1 µmol/l	^79,80^
INCB054329	∼0.1–1	25–50	∼1 µmol/l	^39,119^

C_*max*_ maximum plasma concentration.

### JQ1

The Bradner lab in collaboration with the Structural Genomics Consortium identified a novel thienotriazolodiazepine-based, selective BETi—termed JQ1—which was derived from less potent compounds patented by the Mitsubishi-Tanabe company in 2006 and 2009. Mitsubishi-Tanabe subsequently published data regarding more potent thienodiazepine derivatives in 2016, including compound ‘7f’, which potently inhibits tumour growth at 20 mg/kg.^40^ Differential scanning fluorimetry revealed that, of the 41 human bromodomain-containing proteins that exist, JQ1 binds with the highest affinity to BRD4.^23^ Crystallographical studies demonstrate that this small molecule mimics acetylated lysine and competitively fits into the binding pocket of the BET bromodomains,^23^ forming a hydrogen bond with a conserved asparagine, and shows affinity for both BD1 and BD2. As a result of this tight fit, JQ1 can displace BRD4 from chromatin (as confirmed by fluorescence recovery after photobleaching cell assay and by chromatin immunoprecipitation).^23^ Since the development of JQ1, many more BETi have been developed, including next-generation compounds that are selective for individual bromodomains of BET proteins.^41,42^

The development of JQ1 offered a great opportunity to better understand the biology of BET proteins, validate the oncogenicity of BRD4 and to determine whether BET proteins are bona fide anti-cancer targets. JQ1 showed anti-cancer properties against NMC models, inducing growth arrest and cell differentiation in NMC-derived cell lines, similar to observations from genetic knockdown studies.^23^ Furthermore, JQ1 has also shown significant anti-tumorigenic activity in mouse xenograft models, in which the compound inhibited tumour growth and improved survival rates. RNA interference screening detected a dependency of acute myeloid leukaemia (AML) models on BRD4 expression, and JQ1 treatment led to anti-cancer effects in in vitro and in vivo settings by inhibiting cell proliferation and inducing myeloid differentiation.^43^ Models of many other cancers, including medulloblastoma, breast and lung cancer, also showed an anti-tumorigenic response to JQ1.^44–46^ However, despite its anti-tumour activity, JQ1 has a poor pharmacokinetic profile and low oral bioavailability.^47^ It has a short half-life of only 1 h and the drug often needs to be administered twice per day to induce a therapeutic effect, although optimal dosing varies from model to model. Subsequent improvements in pharmacological properties have resulted in the synthesis of a JQ1 analogue named TEN-010 (JQ2).^48^ Currently, TEN-010 is undergoing clinical trials in patients with AML, myelodysplastic syndrome (MDS) and solid tumours (NCT02308761, NCT01987362).

OTX015 is a small molecule inhibitor of BRD2, BRD3 and BRD4 that is structurally similar to JQ1.^49,50^ One important advance in the development of this inhibitor is its capacity to be administered orally. In preclinical studies, OTX015 showed efficacy against haematological malignancies including B-cell lymphoma, multiple myeloma and some solid types of cancer such as neuroblastoma and mesothelioma.^47,51–53^

The average doses for early BETi such as JQ1, I-BET762, I-BET151 and OTX015 generally ranged between 300 nM to1 µM in vitro and 30–50 mg/kg in murine models of cancer.^23,54–57^ While these compounds differ from next-generation molecules in their pharmacokinetics, it is unclear that preclinical findings using such high doses of drug could predict anti-tumour responses in patients at tolerable doses. In fact, the clinical utility of most BETi evaluated to date has been limited due to unexpected toxicities (described in more detail below). The first clinical trials incorporating BETi tested OTX015 against both haematopoietic and solid cancers (described below). These patients displayed severe dose limited toxicities including gastrointestinal disorders, anaemia, thrombocytopenia, hyperbilirubinaemia, fatigue, headache and back pain.^58–60^ More recently, a clinical trial using a new BETi, BAY1238097, against solid cancers has been terminated because of dose-limiting toxicities.^61^

### I-BET762

In parallel with the development of JQ1, the benzodiazepine I-BET762 was developed by GlaxoSmithKline.^30^ I-BET762 differentiated itself from JQ1 in that it is orally bioavailable, making it amenable to clinical evaluation.^62^ This compound demonstrates a pan-affinity profile against BET proteins, targeting BRD2, BRD3 and BRD4. I-BET762 was initially characterised as a chemical means to reduce inflammation.^30^ By dissociating BET-family members from enhancer regions, I-BET762 downregulates a set of pro-inflammatory genes in activated macrophages in vitro and reduced inflammation in murine sepsis models.^30^ Subsequent to these findings, I-BET762 was tested against a number of preclinical cancer models. Similar to JQ1, I-BET762 showed efficacy against multiple myeloma models in vivo.^55^ Although not as widely studied as JQ1, I-BET762 was shown to be effective in diverse preclinical tumour models including neuroblastoma and pancreatic cancer.^54,63^ As described below, I-BET762 progressed to clinical trial, but a similar spectrum of adverse events to OTX015 were observed, indicating these toxicities may be common across BETi.^62^ Next-generation molecules, described below, aim to overcome these constraints through increasing potency and selectivity.

### Advances in selective BETi design

One approach to achieving greater selectivity and potentially reducing unwanted toxicities is to target only one of the two bromodomains of BET proteins. While most selective BET inhibitors developed thus far target the BD2 domain, there is evidence to suggest that targeting the BD1 domain might be sufficient to elicit anti-proliferative effects,^14^ although this concept remains controversial given the potent tumour suppression seen using BD2-selective inhibitors.^64^ Interpretation of data gathered using BD-selective agents might be tempered based on a recent study that thoroughly tested a panel of 18 BETi for their selectivity against BD1 versus BD2 domains. Of these, only ABBV-744 and two molecules described within the article, GSK778 (iBET-BD1) and GSK046 (iBET-BD2) showed appreciable selectivity. The two novel ‘iBET’ molecules display the highest degree of selectivity described to date. Their potency against one BD versus the other, differs by a concentration of at least tenfold. An intriguing finding of this study was that targeting only BD1 is sufficient to phenocopy the anti-proliferative effects of pan-BETi in vitro.^14^ BD2 was found to be essential for the activation of interferon response genes. GSK778 also displayed strong anti-cancer effects in vivo, prolonging the survival of mice carrying an aggressive form of AML at only 15 mg/kg.

ABBV-744 is highly selective for BD2 of BRD2, BRD3 and BRD4,^64^ exhibiting several hundred-fold higher affinity for the BD2 over BD1.^65^ ABBV-744 shows potent anti-proliferative effects against numerous AML and prostate cancer cell lines, with IC50s in the low nanomolar range. This sensitivity translated well in vivo, with prostate xenografts responding to as low a dose as 4.7 mg/kg, and was associated with a favourable toxicity profile. This is a considerable improvement upon earlier studies using JQ1, where 50–100 mg/kg were commonly employed in vivo. The data for ABBV-744 contrast the reports from Gilan et al, outlined above, indicating that only BD1 is required for anti-proliferative effects of BETi.^14^ To reconcile these differences, further research is required, but is certainly possible that BD1 and BD2 may play unique roles in driving proliferation across different types of tissue.

SJ432 is another recent, rationally designed, BD2-selective BETi.^66^ It is encouraging that BD2-selective molecules, such as SJ432 show greater potency than the prototype BETi JQ1 in vivo, against neuroblastoma models at only 15 mg/kg, indicating that such compounds might retain potency while avoiding some of the unwanted toxicities associated with high doses of BETi. These findings also contrast with the recent publication from the GlaxoSmithKline group^14^ where evidence was presented that targeting BD1 is a primary mechanism whereby BETi achieve growth inhibition.

Moving forward, it will be important for independent groups to repeat the findings of published reports using BD-selective BETi and determine whether targeting BD1 or BD2 will ameliorate the class-specific toxicities of BETi while maintaining their anti-tumour activity.

### Advances in pan-BETi

RVX-208 was one of the first BETi described after the advent of JQ1 and I-BET762 This quinazoline derivative was initially described as a BD2-selective compound, recognising the C-terminal bromodomain of BRD2 and BRD3, inhibiting these proteins with an IC50 of approximately 500 nM.^67^ RVX-208 is currently under clinical evaluation for the treatment of atherosclerosis and other high-density lipoprotein-linked diseases^68–71^ by virtue of its ability to increase the levels of apolipoprotein A1 (ApoA1) and reduce vascular inflammation in manner dependent on its inhibition of BET proteins.^72^

A potent BETi, MS417, which binds to BD1 and BD2 of BRD4 with a dissociation constant (*K*_d_) of 25–36 nM,^73,74^ has been shown to completely inactivate transcription mediated by NF-κB at 1 µM in human embryonic HEK293T cells. In an in vivo breast cancer model, MS417 showed a more pronounced anti-tumour effect at only 20 mg/kg compared with JQ1 used at 50 mg/kg.^75^ MS417 at 20 mg/kg also significantly decreased liver metastasis in colorectal cancer preclinical models.^76^

ABBV-075 (Mivebresib) is an orally bioavailable, pan-BETi with a pyrrolopyridone core, demonstrates exceptional potency against BRD2, BRD4 and BRDT, with an inhibition constant (*K*_i_) of only 1–2.2 nmol/l.^77^ Notably, this small molecule requires tenfold higher concentrations to reduce BRD3 activity to the same degree, although the Ki is still a respectable value of 12.2 nmol/l. It is unclear why this molecule has a lower affinity for BRD3, but is likely due to subtle peptide variances between BRD2 and BRD3 within both BDs.

A large screen of 147 haematological and solid cancer cell lines demonstrated that ABBV-075 inhibits cell proliferation at an IC50 of ~100 nM, with higher efficiency in haematological cancer models than solid tumour models.^77^ These encouraging data propelled this molecule forward for clinical evaluation.^78^ Moving forward, it will be interesting to compare the in vivo toxicity profile of compounds such as ABBV-075, which demonstrate potent inhibition of several family members, with those that are designed to show greater selectivity.

An innovative approach to optimise BETi activity is through synthesis of bivalent BETi, such as AZD5153, designed to simultaneously recognise, and interact with, both bromodomains of BRD4.^79^ AZD5153 enhanced the displacement of BRD4 from chromatin at lower concentrations than did I-BET762, and showed significant inhibitory capacity against cell growth using haematological cancer models (GI50 <25 nM). Importantly, preliminary data using AZD5153 show encouraging results in vitro and in vivo against haematologic and thyroid malignancies at concentrations as low as 5*–*10 mg/kg.^79,80^

## Biomarkers

### Predictive biomarkers

Treatment with BETi has been suggested to be an efficient strategy for multiple preclinical cancer models including NMC, AML, myeloma, lung, breast and pancreatic cancer.^23,43,45,55,81,82^ However, not all cells show sensitivity to the inhibitors, especially at low doses, and it has been challenging to identify the tumour subtypes, or predictive biomarkers thereof, that will show the highest degree of growth inhibition after BETi exposure. This is partially due to variability in defining precise concentrations of BETi that affect ‘sensitive’ cells. Many studies published to date using first-generation compounds have used concentrations that typically range between 500 nM and 1 µM for in vitro studies and ≈50 mg/kg in animal studies.^23,45,55,81,82^ This could make the discovery of robust biomarkers challenging, because biomarkers predicting sensitivity to these concentrations are unlikely to translate to a clinical setting, in which such doses are unattainably high. Thus, there is currently an emerging need to carry out BETi research using lower doses in preclinical experiments in order to properly stratify tumours into responders and non-responders. Such approaches will also help future clinical trials avoid undesirable toxicities. We propose that next-generation BETi showing efficacy in vitro at low nanomolar concentrations will be ideal for biomarker studies.

#### Overexpression of BET-family members

Substantial evidence indicates that the primary target of most BETi, BRD4, is oncogenic—therefore identifying tumour types that are dependent on BRD4 for survival might be one way to identify those tumours that will be most sensitive to BRD4 inhibition. A topic that is underexplored is whether overexpression of BET-family members themselves influences the sensitivity to BETi. Although there are many scenarios, it is possible that tumours that overexpress BRD4 depend on its expression for survival. A caveat here involves the selectivity profile of BETi. Currently, most BETi target all the members of BET subfamily, including BRD2, BRD3, BRD4 and BRDT.^23,30,49,50^ These proteins might have both distinct and overlapping functions, which requires deeper understanding of the mechanism of action, and oncogenic driver activity, of these proteins in different cancers. Exogenous expression of each BET protein in distinct cell types, either alone or in combination, would help to elucidate tissue-specific oncogenic properties of these chromatin-binding proteins and allow their impact on sensitivity to BETi to be examined.

#### MYC amplification

**Fig. 2:**
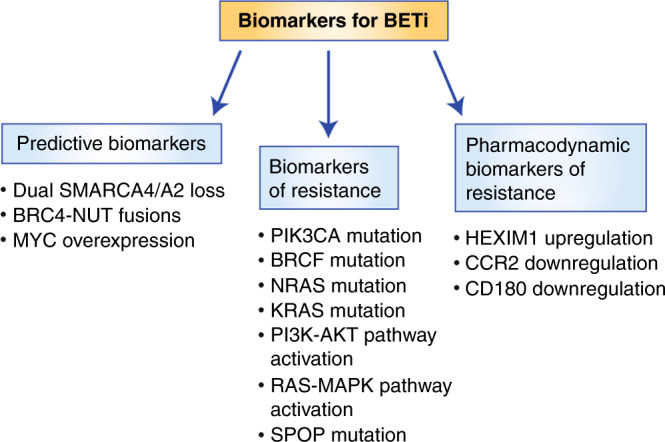
Biomarkers for BETi. Three types of biomarkers to BETi treatment exist: predictive biomarkers, biomarkers of resistance and pharmacodynamic biomarkers.

A key discovery regarding the anti-tumour activity of BETi is that these drugs, including first- and second-generation compounds, silence the expression of *MYC* in preclinical studies, and it appears that the amplification of *MYC* family members predicts sensitivity to BETi in multiple tumour types.^19,64,66^ It was therefore widely predicted that elevated levels of *MYC* would enhance sensitivity to BETi (Fig. 2). However, although *MYC* is commonly downregulated in cells exposed to BETi, it is not always clear that this event mediates the anti-proliferative effects of BETi, and several studies have shown that *MYC* does not always act as a mediator of BETi sensitivity.^83–86^ Many of the studies reporting *MYC* as a principal factor in the sensitivity to BETi were carried out in models of haematological malignancies; however, initial clinical studies using BETi directed against multiple myeloma, AML and diffuse large B-cell lymphoma (DLBCL) found that *MYC* amplification failed to predict sensitivity to the BETi OTX015.^58,59^ A 2020 clinical trial found that, although the levels of MYC in peripheral blood samples of patients with castrate-resistant prostate cancer were reduced after exposure to the pan-BETi Zen-3694, correlations between reduced MYC levels in peripheral blood and patient responses could not be established; intratumoral *MYC* was not examined.^87^ These studies question the validity of using the MYC protein as a predictive biomarker. It is possible that high concentrations of BETi used in preclinical studies have off-target effects that are either exacerbated in MYC-expressing cells, or act through BET-independent pathways to silence MYC. Again, these concerns highlight the need to carry out preclinical studies at relevant concentrations, not far in excess of the IC50 required to dissociate BET proteins from chromatin. To establish *MYC* amplification as a robust predictive biomarker, clinical studies demonstrating significant patient responses in *MYC*-amplified patient cohorts, coupled with a downregulation of *MYC* in response to drug exposure, would be required. Clinical data, described below, suggest that *MYC* suppression holds promise as a pharmacodynamic biomarker.

#### Dual SMARCA4/A2 loss

Understanding mechanisms through which BRD4-driven transcriptional programmes are regulated is likely to reveal vulnerabilities that could be exploited using BETi. BRD4 and another bromodomain protein, SMARCA4, independently but concurrently activate gene expression by simultaneously binding to different regulatory elements in their target genes.^6^ SMARCA2, a SMARCA4 paralogue, is likely to compensate for SMARCA4 loss so that, in cancers harbouring SMARCA4/A2 deficiency, a common underlying molecular lesion, BRD4 might act as the primary driver of SMARCA4/BRD4-dependent oncogenes; consequently, exposure to BETi might eliminate this network to promote cell cycle arrest or apoptosis. Consistent with this hypothesis, our data demonstrated that SMARCA4/A2-deficient small cell cancer of the ovary hypercalcemia type (SCCOHT) and non-small cell lung cancer (NSCLC) models are acutely sensitive to BETi at low nanomolar concentrations in vitro as well as in vivo (20 mg/kg per day).^88^ Further support for this premise comes from the observation that ectopic expression of SMARCA4 was found to confer partial resistance to BETi. These findings demonstrate that the loss of SMARCA4 might act as a potential predictive biomarker for the effectiveness of BETi (Fig. 2). Notably, in OTX015-sensitive cells that are deficient in SMARCA2 and SMARCA4, repression of the extracellular signal-regulated kinase (ERK)/mitogen-activated protein kinase (MAPK) pathway was tightly correlated with drug efficacy. Again, this suggests that the RAS–ERK/MAPK pathway plays an important role in dictating the response to BETi.

BETi inhibitors act primarily through the repression of oncogenic networks, including those regulated by MYC protein levels. It should therefore not be surprising that in some cancers, such as AML and triple-negative breast cancer, epigenetic reprogramming to maintain the expression of these oncogenic networks is a mechanism of both intrinsic and acquired resistance.^89,90^ In particular, there is substantial evidence that changes to the epigenetic landscape mediated by Polycomb group proteins might dictate responses to BETi.^91,92^ Future work will provide insights into the potential use of the expression profiles of Polycomb group proteins as biomarkers and the potential synergy between inhibitors of the Polycomb group protein EZH2 and BETi.

### Biomarkers of resistance

#### Constitutive activation of receptor tyrosine kinase (RTK) signalling cascades

RTK pathways mediate proliferation and survival through the activation of downstream phosphatidylinositol 3-kinase (PI3K)–AKT and RAS–ERK/ MAPK signalling cascades. Many types of cancer are characterised by increased transduction through these classical oncogenic networks. Beyond promoting their effects on sustained tumour growth, it has become clear that both the PI3K and ERK/MAPK axis mediate acquired resistance to broad categories of anti-cancer agents.^93^ The first evidence that RTK signalling may be involved in both intrinsic and acquired resistance to BETi came from unexpected observations in sensitive cells. Notably, numerous studies found that BETi inactivate transcriptional programmes involved in RTK signalling exclusively in sensitive cells.^88,94–98^ Supporting this concept, the activation of PI3K and ERK/MAPK signalling cascades appears to dictate intrinsic resistance to BETi in multiple types of cancer (Fig. 2).^88,97–100^ This was clearly demonstrated through ectopic expression of constitutively active downstream effectors of these pathways, including AKT, KRAS and BRAF, all of whom conferred resistance to BETi.^99,101^

CRISPR/Cas9 screening in a neuroblastoma model revealed that PI3K activation imparts resistance to BETi, and such activation might play a role in either intrinsic or acquired resistance to BETi.^96^ Here, a large-scale screening study using complementary technologies, including ORF rescue, CRISPR/Cas9 rescue and models of in vitro acquired resistance to BETi, revealed that enhancer remodelling to facilitate upregulation of RTK and PI3K signalling is a common mechanism of BETi resistance.^96^ Specifically, transcriptional activation of resistance genes was nearly always associated with a strong enrichment for the histone modification H3K27Ac at enhancer elements. On nearly half of these active enhancers, an enrichment for BRD4 binding was concurrently observed. Notably, based on understanding this underlying mechanism of resistance, it was revealed that these neuroblastoma cell models were sensitive to PI3K inhibitors in combination with BETi.

Likewise, ovarian cancer cells acquiring BETi resistance through long-term culture reprogrammed their kinome to elevate signalling through PI3K and RAS pathways.^98^ Interestingly, increased RTK signalling appeared to be mediated by transcriptional upregulation of mitogenic ligands. However, exome sequencing was not carried out, so de novo activating mutations cannot be excluded.

In colorectal cancer models, *KRAS* mutations do not appear to render tumour cells more resistant to BETi.^102^ These cells harbour a high degree of intrinsic resistance to BETi regardless of their mutational profile. In *KRAS*-mutant-carrying NSCLC models, sensitivity to BETi was reported, but 2.5 µM JQ1 was required to induce anti-proliferative responses, meaning that, in reality, these cells are quite resistant to BETi. *MYC* downregulation was observed at 500 nM JQ1, which is insufficient to elicit an anti-proliferative effect, indicating that *MYC* downregulation alone could not prompt cells to stop dividing.^82^ Notably, this study revealed that the loss of the LKB1 tumour suppressor, an upstream suppressor of mechanistic target of rapamycin (mTOR) activity, led to BETi resistance. mTOR is a key downstream effector of the PI3K pathway, regulating diverse processes including protein synthesis and cytoskeletal remodelling. In this model, loss of LKB1 is expected to increase mTOR signalling thereby imparting resistance to BETi.

Together, these data suggest that gain-of-function mutations and constitutive activity through the PI3K and RAS signalling axes might act as biomarkers of resistance to BETi in specific types of cancer (Fig. 2). As described below, this information points towards the use of rational combination therapies to overcome this resistance.

#### SPOP mutation

The stability of BET proteins might also influence their susceptibility to inhibitors. Speckle-type POZ protein (SPOP), which is responsible for the ubiquitination and degradation of BET proteins, is found mutated in 6–15% of prostate tumours across different case series.^103^ In prostate cancer, mutations within the substrate recognition domain (SRD) of SPOP confer intrinsic resistance to BETi both in vitro and in vivo, but depletion of BET proteins re-sensitises cells to this treatment (Fig. 2).^104,105^ BET proteins are direct targets of SPOP and the mechanism underlying BETi resistance in *SPOP*-mutated cells is a decreased rate of BET protein degradation resulting in the accumulation of BET-family members.^106^ Evidence suggests that a combination BRD4/CBP inhibitor (NEO2734) overcomes SPOP resistance.^107^ In SPOP-mutated prostate cancer the androgen receptor (AR) is quite active and critical for tumour survival. CBP/p300 acts as an essential AR co-activator, and disrupting its function is probably a major mechanism whereby NEO2734 works in this system. Remarkably, in endometrial cancer, some SPOP SRD mutations, such as R121Q, lead to an enhanced association between SPOP and BET-family members, which results in increased ubiquitylation, decreased BET protein levels and enhanced sensitivity to BETi.^106^ This underscores the importance of understanding the nature of mutations in their appropriate biological context. Overall, SPOP mutations hold great promise as biomarkers of both resistance and sensitivity to BETi.

These studies reinforce the notion that BRD4 levels within tumours modulate the responsiveness to BETi. The data also suggest that the activity of BET proteins might be regulated by proteasomal degradation across tissue types, which opens new therapeutic perspectives. Especially relevant might be targeting BET proteins, either individually or combinatorially, using a proteolysis targeting chimera (PROTAC, reviewed in ref. ^108^).

### Pharmacodynamic biomarkers

Pharmacodynamic biomarkers enable the clinical monitoring of drug activity in vivo, ideally using simple PCR or ELISA-based assays that act in lieu of more complex approaches to measure the accumulation of an administered drug within plasma or the tumour itself. Identifying robust pharmacodynamic biomarkers for BETi in preclinical settings will undoubtedly facilitate the optimisation of their clinical utility. Generating such biomarkers might be enhanced through the preclinical use of orthotopic xenografts that mimic a relevant tumour microenvironment, thereby more accurately predicting drug response in a time- and dose-dependent manner.

#### HEXIM1 upregulation

Upregulation of HEXIM1 has been identified as a potential pharmacodynamic biomarker in numerous tumour xenografts and whole blood samples in response to ABBV-075 treatment (Fig. 2).^109^ HEXIM1 inhibits P-TEFb by sequestering it into an inactive complex; accordingly, upregulation of HEXIM1 leads to the inhibition of transcriptional elongation by RNA polymerase II.^110^ This represents an indirect mechanism through which BETi could repress transcription. A small clinical trial showed a trend for both *MYC* repression and an increase in HEXIM1 in the plasma of patients treated with the BETi BAY1238097 for mixed, refractory malignancies.^61^ This trend was observed when plasma concentrations of the BETi reached approximately 20 µg/l or greater. This plasma concentration could be achieved at clinically obtaining doses of 40 mg/week. This is below administered drug levels eliciting dose-limiting toxicities. Of interest, while 40 mg/week represents an exposure capable of lowering *MYC* levels, tumour repression was not observed at this dose. This may cast some doubt on the necessity for BETi to lower *MYC* transcription in order to provoke an anti-tumour response.

Another preclinical study reported that *MYC* and HEXIM1 act most robustly as tumour-based pharmacodynamic biomarkers for BETi and that the corresponding mRNAs did not show optimal correlation with drug accumulation within blood samples.^111^ Thus, *MYC* modulation (repression) in particular might hold more promise as an indicator of drug activity than its amplification does in predicting drug response.

#### CCR2 and CD180 downregulation

The study from Yeh et al., also discovered that the levels of C-C chemokine receptor type 2 (CCR2) and CD180 were found to be significantly downregulated in whole blood samples exposed to AZD5153 from patients with multiple types of cancer (Fig. 2).^111^ Originally identified as BETi targets using transcriptomic analysis, the downregulation of these immune components might link BETi activity with the modulation of immune infiltrates. This is especially relevant for CCR2, that is known to recruit myeloid-derived suppressor cells to the tumour niche, which in turn, promotes tumour growth.^112,113^ Further clinical trials are required to validate these findings and to determine whether patients responding to BETi show changes in the number of tumour-infiltrating myeloid cells.

## Lessons from clinical trials

After extensive preclinical evaluation, but in the absence of robust predictive biomarkers, multiple clinical trials of BETi against solid and haematological types of cancer have been initiated. To date, ~25 clinical trials are either ongoing or have been completed (Table 2). With the exception of NMC, these trials are not targeting specific molecular subtypes, and this lack of biological rationale, plus unexpected toxicities, might limit clinical efficacy in the short term.

**Table 2:** Clinical trials with BET inhibitors.

BET inhibitor	Reference	Sponsor	Combination	Phase/status	Indications	Results
FT-1101	NCT02543879	Forma Therapeutics, Inc.	Azacitidine	Phase 1, completed	Haematological malignancies	Not posted
RO6870810	NCT03068351	Hoffman-La Roche	Daratumumab	Phase 1, completed	Advanced multiple myeloma	Not posted
CPI-0610	NCT02157636	Constellation Pharmaceuticals	N/A	Phase 1, completed	Multiple myeloma	Not posted
CPI-0610	NCT01949883	Constellation Pharmaceuticals	N/A	Phase 1, completed	Lymphoma	CR: 2 PR: 1 *n* = 44
CPI-0610	NCT02158858	Constellation Pharmaceuticals	Rixolitinib	Phase 1/2, recruiting	Haematological malignancies, myelofibrosis	Not posted
CPI-0610	NCT02986919	Texas Southwestern Medical Center	N/A	Phase 2, withdrawn	Peripheral nerve tumours	Not posted
I-BET762 (molibresib)	NCT01943851	GlaxoSmithKline	N/A	Phase 1/2, completed	Haematological malignancies	Not posted
I-BET762 (molobresib)	NCT03266159	GlaxoSmithKline	Trametinib	Phase 1/2, withdrawn	Solid tumours	Not posted
I-BET762 (molibresib)	NCT01587703	GlaxoSmithKline	N/A	Phase 1/2, active, not recruiting	NMC	PR: 2 *n* = 17
ZEN003694	NCT02711956	Zenith Epigenetics	Enzalutamide	Phase 1/2, active, not recruiting	Prostate cancer	Not posted
ZEN003694	NCT02705469	Zenith Epigenetics	N/A	Phase 1, completed	Prostate cancer	Not posted
INCB054329	NCT02431260	Incyte Corporation	N/A	Phase 1/2, terminated	Solid and haematological malignancies	PR: 1 *n* = 54
BMS-986158	NCT02419417	Bristol-Myers Squibb	Nivolumab	Phase 1/2, recruiting	Advanced solid tumours and haematological malignancies	Not posted
MK-8628 (OTX015)	NCT02303782	Oncoethix GmbH	Azacitidine	Phase 1/2, withdrawn	AML	Not posted
MK-8628 (OTX015)	NCT02698189	Merck Sharp & Dohme Corp.	N/A	Phase 1, active, not recruiting	Haematological malignancies	NR *n* = 9
MK-8628 (OTX015)	NCT02698176	Merck Sharp & Dohme Corp.	N/A	Phase 1, terminated (limited efficacy)	Advanced solid tumours	NR *n* = 13
MK-8628 (OTX015	NCT02296476	Oncoethix GmbH	N/A	Phase 2, terminated	Glioblastoma multiforme	NR *n* = 12
MK-8628 (OTX015)	NCT02259114	Oncoethix GmbH	N/A	Phase 1, completed	Advanced solid tumours	PR (NMC): 3 *n* = 46
MK-8628 (OTX015)	NCT01713582	Oncoethix GmbH	N/A	Phase 1, completed	Haematological malignancies	CR (DLBCL):2 PR (DLBCL):1 *n* = 33; CR (AL): 2 PR (AL): 3 *n* = 41
RO6870810/TEN010	NCT02308761	Hoffmann-La Roche	N/A	Phase 1, completed	Haematological malignancies	Not posted
RO6870810/TEN010	NCT01987362	Hoffmann-La Roche	N/A	Phase 1, completed	Solid tumours	PR (NMC): 2 *n* = 3
BAY1238097	NCT02369029	Bayer	N/A	Phase 1, terminated	Solid tumours, non-Hodgkin lymphomas	NR *n* = 8
ABBV-744	NCT03360006	AbbVie	N/A	Phase 1, recruiting	AML	Not posted
ABBV-744	NCT04454658	AbbVie	Rixolitinib, Navitoclax	Phase 1, not yet recruiting	Myelofibrosis	Not posted
ABBV-075	NCT02391480	AbbVie	Venetoclax	Phase 1, completed	Solid tumours, AML, multiple myeloma	NR (solid tumours) *n* = 84 CR: 1 (AML) *n* = 41
ABBV-075	NCT04480086	AbbVie	Rixolitinib, Navitoclax	Phase 1, not yet recruiting	Myelofibrosis	Not posted
RVX000222	NCT01728467	Resverlogix Corp	N/A	Phase 2, completed	Pre-diabetes	Not posted
RVX000222	NCT01058018	Resverlogix Corp	N/A	Phase 2, completed	Coronary artery disease	ApoA-I increase up to 5.6% HDL-C increase up to 8.3% *n* = 299
RVX000222	NCT00768274	Resverlogix Corp	N/A	Phase 1, 2 completed	Cardiovascular disease, atherosclerosis	Not posted
RVX000222	NCT02586155	Resverlogix Corp	Atorvastatin, Rosuvastatin	Phase 3, active, not recruiting	Cardiovascular disease, type 2 diabetes	No significant change of primary end point *n* = 2425
RVX000222	NCT01863225	Resverlogix Corp	Atorvastatin, Rosuvastatin	Phase 2, terminated	Dyslipidaemia	Not posted

*CR* complete response, *NMC* NUT midline carcinoma, *PR* partial response, *AML* acute myeloid leukaemia, *NR* no response, *DLBCL* diffuse large B-cell lymphoma, *ApoA-I* apolipoprotein A-I, *HDL-C* high-density lipoprotein-cholesterol.

Strong results from the preclinical evaluation of BETi in multiple myeloma mled to elevated expectations for achieving clinical responses in this malignancy. The first published clinical trials of BETi all used the widely studied drug OTX015 (MK-8268).^58–60,114^ In one of these Phase 1 trials, OTX015 was tested in 45 patients (33 with lymphoma and 12 with myeloma): two patients with DLBCL showed a complete response and another with DLBCL responded partially to the treatment,^58^ but, unfortunately, there were no reports of patients with multiple myeloma who displayed responsiveness. Among five patients with MYC-positive DLBCL, only one showed a favourable outcome to BETi exposure. These studies established a drug dose of 80 mg/day for a schedule of 14 days on, 7 days off (21-day regimen). However, this dose might not be universally achievable because almost all of the patients in this trial displayed dose-limiting toxicities, including thrombocytopenia, anaemia, neutropenia, gastrointestinal events and fatigue. Based on published clinical data, these toxicities appear to be common for this class of drugs regardless of the type of cancer being treated or the chemical structure of the BETi being tested.^62,115^ Again, these studies highlight the importance of identifying pharmacodynamic biomarkers for BETi, which could help guide dose-escalation studies. Once maximum drug activity is identified in the plasma or intratumorally, higher doses are likely to elicit strong off-target effects.

As described above, NMC is characterised by BRD4 fusions leading to aberrant BRD4 activity. Again, based on preclinical data, expectations that BETi would show efficacy against these tumours were high and, consequently, the first published clinical trial using BETi was focused on NMC.^114^ In this trial, a small series of four patients carrying diverse, advanced-stage tumours harbouring BRD4–NUT fusions received OTX015. Clinical responses were observed in two patients, but all four patients succumbed to their disease between 5 and 19 months post-diagnosis. Complementing this study, Lewin et al.^60^ observed partial responses in three out of ten patients with NMC receiving OTX015 at 80 mg once daily, for a duration of 1.8–8.4 months. Partial responses have also been described for NMC patients treated with the I-BET762A (molibresib).^62^ While somewhat encouraging, these trials have not met expectations, and the reason for the discrepancy between preclinical observations and clinical results remains obscure.

In addition to MYC and BRD4–NUT-driven malignancies, BETi have been tested against several AMLs and diverse solid tumours. In an initial study of OTX015 in a cohort of 41 patients with AML,^59^ three patients showed partial responses and two patients showed a complete response lasting 2–5 months. Notably, however, an attempt to identify potential biomarkers including mutations in 42 genes failed to uncover any clear molecular pathologies among responders. Although published reports of the use of BETi against solid tumours have not met expectations, responses have been observed, again underscoring a need for predictive biomarkers. Perhaps the most promising preclinical data against solid tumours beyond NMC have come from prostate cancer models.^60^ However, 26 patients harbouring castration-resistant prostate cancer (CRPC) showed little response to OTX015 with either continuous or discontinuous regimens in clinical trials.^60^ Similar data were acquired testing I-BET762 against nine CRPC patients.^62^ Perhaps surprisingly, a partial response was observed in a breast cancer patient in this Phase 1 study. This study included patient cohorts harbouring both solid and haematological tumours. In total, 65 patients were enrolled, including those with NMC, small cell- and non-small lung cancer, triple-negative breast cancer (TNBC), CRPC, colorectal cancer, neuroblastoma and multiple myeloma. While complete responses were not reported, four NMC patients had confirmed and unconfirmed partial responses and one TNBC patient achieved unconfirmed partial response.

Based on these data, we predict that BETi might play an important clinical role in the management of NMC. However, it is clear that predictive biomarkers and, most likely, combinatorial approaches, will be essential moving forward and, perhaps more importantly, it will be critical to overcome in-class dose-limiting toxicities. BET proteins are important for multiple cellular processes required for homoeostasis, which might explain why complete bromodomain inhibition leads to unexpected toxicities. Advances in the medicinal chemistry of BETi might be required to dissociate anti-cancer effects from the inhibition of physiological pathways required for homoeostasis.

### Combination therapies

Preclinical research demonstrating the efficacy of BETi as a single agent often uses drug concentrations of 500 nM to micromolar concentrations and in vivo concentrations that are unattainable in a clinical setting. The necessity of using such high doses to combat cancer proliferation strongly suggests a high level of intrinsic resistance to these compounds across tumour types that has not been widely studied or appreciated. The precise mechanisms of intrinsic resistance to BETi remain unclear, but multiple studies suggest that the activation of oncogenic signalling pathways including those mediated by PI3K and RAS might be involved and that repression of these pathways might act as key mediators of BETi activity. We suggest that, consequently, these pathways also play a role in intrinsic BETi resistance and could therefore be targeted in conjunction with the use of BETi.

Several studies report the PI3K pathway as a determining factor influencing BETi both intrinsic and acquired resistance, and suggest that this resistance could be successfully overcome through the combination treatment using BETi and PI3K inhibitors.^95,96,98^ *PIK3CA* is one of the most frequently mutated genes in cancer, with *PIK3CA* mutations occurring in ~30% of patients with breast, endometrial and colorectal cancer.^116–118^ *PIK3CA*-mutated breast cancer models are intrinsically resistant to treatment using either PI3K inhibitors or BETi individually.^94,95^ Likewise, transcriptional upregulation of RTK-related genes through BRD4-driven chromatin remodelling of enhancer regions might also mediate acquired BETi resistance.^96^ Clearly, understanding the interplay between BRD4 and signalling pathways will help us optimise the use of BETi as anti-cancer agents and avoid use against tumours identified as intrinsically resistant. As described below, a better understanding the mechanisms of resistance mediated by constitutive signalling through PI3K and MAPK pathways will probably reveal new therapeutic options for these patients.

#### Combined treatment with BETi and inhibitors of oncogenic signalling pathways

Multiple studies demonstrate that combining BETi with tyrosine kinase inhibitors effectively overcomes both intrinsic and acquired resistance to these drugs in several types of cancer.^94,97,98^

A chemical combinatorial screening of JQ1 with around 1900 compounds aimed at finding effective small molecule combination therapies with BETi revealed PI3K inhibitors to be the most potent partners against neuroblastoma both in vitro and in animal models.^96^ The combination of PI3K inhibitors and BETi also proved to repress the proliferation of ovarian cancer cells with acquired resistance to BETi.^98^ It remains unclear precisely how BETi enhance the effects of PI3K inhibitors—perhaps it stems from transcriptional repression of RTKs themselves, or their downstream effectors.

Considering what we know thus far regarding BETi resistance, and given evidence from preliminary studies, it is logical to predict that BETi might act in synergy not only with inhibitors of PI3K but also with inhibitors of components of the Ras–ERK/MAPK signalling pathway, such as MAPK and ERK kinase (MEK), to overcome intrinsic resistance. Melanomas bearing *NRAS* mutations frequently have high BRD4 mRNA and protein expression levels, which are associated with a poor outcome.^97^ Such cells display a weak response to the relatively high concentration of BETi of 500 nM. By contrast, the combination of BETi and 100 nM of the MEK inhibitor PD901 showed efficacy in inhibiting cell proliferation, which was validated in mouse models, where a synergy between the two agents was observed.^97^ In vivo, synergy was observed in patient-derived xenograft (PDX) models using only 25 mg/kg OTX015 in combination with another MEK inhibitor, PD0325901. The combined treatment downregulated proteins implicated in cell cycle regulation and apoptosis. Recent evidence indicates that cell cycle regulation resulting from the combination of BETi with MAPK inhibitors may stem from a broad repression of nucleotide metabolism in ovarian cancers.^99^ This was associated with synergistic, in vivo, repression of enzymes involved in nucleotide metabolism, such as RRM1, DUT and TYMS. Moving forward, it will be important to examine whether this mechanism is relevant to other cancer models sensitive to this combination.

About half of all recurrent TNBC express high levels of n-MYC.^119^ These n-MYC overexpressing cells appear to be quite sensitive to the combination of the MEK inhibitor trametinib with high dose (500 nM) JQ1. This combination effectively repressed the levels of both *n-MYC* and *MYC* as well as ERK/MAPK phosphorylation. It remains to be experimentally proven which, if any, of these correlates are responsible for the observed growth inhibition. In PDX models of TNBC, intermediate or high levels of n-MYC predicted response to the synergistic combination of trametinib with either JQ1 or another BETi, INCB054329, whereas PDX models with low levels of n-MYC were more resistant. As stated above, the half-life of JQ1 in vivo is quite short,^120^ and high doses are generally required to achieve effects. Unfortunately, the levels of BETi typically required, such as those used in this study—50 mg/kg twice daily—are quite disconnected from clinically achievable concentrations of BETi, and it is unclear whether such preclinical data strongly predict clinical success. It is likely that next-generation, selective inhibitors that show anti-tumour activity at less than 10 mg/kg will more accurately predict clinical success.^64^

#### Other BETi combinatorial approaches

Additional combinations using BETi also show promise. *KRAS* mutations are very prevalent in pancreatic ductal adenocarcinoma (PDAC) and is associated with a very poor prognosis.^121^ One study showed that the presence of a *KRAS* mutation correlated with the overexpression of BET proteins including BRD2, BRD3 and BRD4 in PDAC.^81^ In this scenario, JQ1 and histone deacetylase (HDAC) inhibitors were found to act in synergy in vivo. Again, however, the doses of drugs employed in this study were 50 mg/kg per day, adding uncertainty to the translation of this combination to a clinical setting.

Mechanistically, this combination is challenging to rationalise, as it would be expected that HDAC inhibition would increase global histone acetylation levels, providing a platform for BRD2/3/4 binding. That being said, independent reports have supported the applicability of this combination in vivo, using either a MYC-driven lymphoma model or against a neuroblastoma model.^122,123^ Mechanistic insights from these studies were lacking, but based on gene expression profiles of neuroblastoma cells treated with BETi+HDACi, a large panel of genes are regulated in a synergistic manner, including MYC transcriptional networks.

As stated previously, BET proteins might act as oncogenes, at least in part, through the activation of genes involved in cell cycle progression. As such, it would be logical to combine BETi with inhibitors of CDKs. This approach has been explored successfully in NMC.^124^ An impressive screen of over 3900 compounds for those showing enhanced efficacy in TNBC cells with acquired resistance to BETi indicated that CDK4/6 inhibitors might be used for this purpose.^90^ It remains to be seen whether such inhibitors might be used to overcome intrinsic resistance.

An interesting combination of BETi with vitamin C was demonstrated to be effective using both in vitro and in vivo TNBC models. In this study, simultaneous treatment resulted in a decrease in histone acetylation,^125^ perhaps consistent with the findings of efficacy between BETi and HDAC inhibitors. This strategy of combining BETi with vitamin C not only in TNBC, but also in melanoma significantly improved the EC50 of BETi by reducing it to a nanomolar range.^125,126^

Even though responses differ across tissue types, the cell response to BETi is generally cytostatic in nature, resulting in delayed cell cycle progression. Thus, it might be logical to complement this cytostatic response with promoters of an apoptotic response. Supporting this idea are a number of studies showing synergy between several BETi and the BCL-2 inhibitor venetoclax (ABT-199) against haematopoietic malignancies.^127,128^ Further work will be required to define the precise mechanism by which these inhibitors act in synergy.

The combination of inhibitors of poly-ADP ribose polymerase (PARP) with BETi has shown great promise, instigating a loss of proliferation across many cell types, including ovarian, breast and pancreatic cancer models both in vitro and in vivo.^129–132^ Mechanistically, inhibition of BRD4 leads to decreased levels of the resection protein C-terminal binding protein-interacting protein (CtIP), which mediates a requisite step in the repair of DNA double-strand breaks through the homologous recombination repair pathway, thereby rendering cells sensitive to PARP inhibition.^129^ The synergy between BETi and PARPi appears to be independent of defects in *BRCA1/2*, *TP53* or *KRAS*, suggesting this approach might have widespread clinical potential.^131^

## Conclusions

The development of BETi has provided important insights into the key role of BET proteins in the transcriptional control of proto-oncogenes, and highlighted the potential of these proteins as therapeutic targets. Preclinical studies have demonstrated a remarkable anti-proliferative activity of BETi against tumours, including several incurable subtypes. It is our opinion that a lack of biomarkers predicting sensitivity to BETi, coupled with the use of non-clinically relevant doses in preclinical studies, is limiting the application of these agents in clinical practice. Further research and mechanistic studies will help to identify such biomarkers, and the development of novel, highly selective bromodomain inhibitors will help prevent toxicities. Finally, exciting new data indicate that, in the long term, BETi are likely to hold the most clinical potential as a part of combinatorial regimens.

## Data Availability

Not applicable.
